# Temporal trend and epidemiological profile of notifications of violence against women in Brazil: 2014-2023

**DOI:** 10.1590/S2237-96222025v34e20240475.en

**Published:** 2025-08-08

**Authors:** Geovanna Carvalho Cardoso Lima, Camila Mendes dos Passos, Alba Lúcia Santos Pinheiro, Ícaro José Santos Ribeiro, Emanuella Gomes Maia

**Affiliations:** 1 Universidade Estadual de Santa Cruz, Programa de Pós-Graduação em Enfermagem e Saúde da Universidade Federal da Bahia, Salvador, Bahia, Brazil; 2 Universidade Federal de Viçosa, Departamento de Medicina e Enfermagem, Viçosa, Minas Gerais, Brazil; 3 Universidade Estadual de Santa Cruz, Departamento de Ciências da Saúde, Ilhéus, Bahia, Brazil; 4 Universidade Estadual de Santa Cruz, Programa de Pós-Graduação em Enfermagem, Ilhéus, Bahia, Brazil

**Keywords:** Violence, Women, Health Information Systems, Time Factors, Public Health, Violencia, Mujeres, Sistemas de Información de Salud, Factores de Tiempo, Salud Pública

## Abstract

**Objective:**

To analyze the temporal trend and epidemiological profile of notifications of violence against women in Brazil between 2014 and 2023.

**Methods:**

This is a time series, conducted using the National System of Injuries and Notifications (SINAN) for interpersonal and self-inflicted violence. Descriptive statistics and Prais-Winsten regression were used to analyze the data.

**Results:**

Notifications of violence against women showed an increasing trend between 2014 and 2023, especially after 2020 (from 9.8 to 19.2 cases per 100,000 women per year). Proportionally, self-inflicted injuries stood out over interpersonal aggression. The majority of notifications referred to adult women (54.0%), with incomplete elementary school from the 4th to the 8th grade (17.4%) and of brown race/skin color (43.0%). Bodily force/beating prevailed as a means of aggression (31.9%), carried out by the spouse (17.3%) and in the home environment (73.4%).

**Conclusion:**

Violence against women has increased in the last decade, with such increase intensifying in recent years. Socially vulnerable women were most affected, especially in cases of sexual violence and neglect/abandonment.

## Resumen

**Objetivo:**

Analizar la tendencia temporal y el perfil epidemiológico de las denuncias de violencia contra la mujer en Brasil entre 2014 y 2023.

**Métodos:**

Se trata de una serie temporal, realizada a partir del Sistema de Información de Lesiones Denunciables de Violencia Interpersonal y Autoinfligida. Se utilizaron estadísticas descriptivas y regresión de Prais-Winsten para analizar los datos.

**Resultados:**

Las denuncias de violencia contra la mujer mostraron una tendencia creciente entre 2014 y 2023, especialmente después de 2020 (de 9,8 a 19,2 casos por cada 100.000 mujeres por año). Proporcionalmente, las lesiones autoinfligidas predominaron sobre la agresión interpersonal. La mayoría de las notificaciones se referían a mujeres adultas (54%), de 4º a 8º grado de primaria incompleta (17,4%) y de raza/color de piel pardo (43%). La fuerza física/golpes prevaleció como medio de agresión (31,9%), realizada por el cónyuge (17,3%) y en el ámbito doméstico (73,4%).

**Conclusión:**

La violencia contra la mujer ha aumentado en la última década, intensificándose este incremento en los últimos años. Las mujeres en situación de vulnerabilidad social fueron las más afectadas, especialmente en casos de violencia sexual y abandono/negligencia.

**Ethical aspects**

This research used public domain data and anonymized databases.

## Introduction

The social roles established since ancient times, such as the submission of females, reflect the cultural, economic and political complexity that involves violence against women in the world and in Brazil. According to the World Health Organization, in 2018, three out of every ten women aged 15 or over were subjected at least once to episodes of physical and/or sexual violence by male aggressors, regardless of whether they had an emotional bond with the victim (1). In Brazil, in turn, according to data from the 2022 Atlas of Violence, more than 50 thousand murders of women were registered between 2009 and 2019, with the main victims being black women (2). 

Violence against women is a serious public health problem and a violation of human rights to which these women are subjected daily. The Belém do Pará Convention of 1994 defined violence against women as any conduct or action based on gender, which causes sexual, psychological or physical suffering, harm or death to women, in the private and public spheres (3). This definition covers everything from self-inflicted/self-harm (against oneself) to interpersonal violence, such as domestic/intrafamily violence (which occurs between intimate partners and family members, mainly, but not exclusively in the residential environment), and extrafamily/community violence (which occurs in the social environment, generally between acquaintances or strangers) (4).

In this context, the country’s surveillance systems are highlighted as important instruments for recognizing the health profile, exposure and risk conditions of the population (5). The violence and accident surveillance system is made up of two components: National System of Injuries and Notifications (SINAN) for interpersonal and self-inflicted violence and Surveillance of violence and accidents in emergency units. The National System of Injuries and Notifications (SINAN) for interpersonal and self-inflicted violence, of interest to this research, is part of the country’s continuous surveillance through compulsory notification of interpersonal and self-inflicted violence. Compulsory notification is characterized as mandatory communication to the health authority about the occurrence of suspected or confirmed disease, injury or public health event (4). In 2003, Law No. 10,778 was published to establish this notification in national territory (6), and in 2004, Ordinance No. 2,406 approved the respective instruments, protocols and flows (7). 

Although data from the National System of Injuries and Notifications (SINAN) for interpersonal and self-inflicted violence have been used in the conduction of some studies, most of them were limited to analyzing one type of violence (8,9), or a specific area of the country (10,11). In this sense, with the aim of contributing to the evidence scenario on violence against women, the objective of this work was to analyze the temporal trend and epidemiological profile of notifications of violence against women in Brazil between 2014 and 2023. It is believed that these notifications have increased in the last decade, especially after the COVID-19 pandemic, and that they have mainly affected the most socioeconomically vulnerable women. 

## Methods

### Study design

This is a time series, retrospective study, with a quantitative approach, guided by the Strengthening the Reporting of Observational Studies in Epidemiology (STROBE) checklist. 

### Participants and data source

The study population was formed by the sum of notifications of violence against women in Brazil between 2014 and 2023. The database for carrying out this research was the National System of Injuries and Notifications (SINAN) for interpersonal and self-inflicted violence. This system is fed by information present in the notification form, which in turn must be filled out and registered by all public health bodies, associated and contracted entities, in the event of any suspected or confirmed case of violence against women, regardless of the victim’s age (4).

Data collection was carried out between November and December 2024 on the website of the Department of Information Technology of the Unified Health System, of the Brazilian Ministry of Health, through the link <https://datasus.saude.gov.br/>. This is publicly accessible secondary data, with guaranteed confidentiality and anonymity for all participants associated with the notifications. The study is in line with the recommendations of the Brazilian National Health Council Resolution No. 466/2012, with no need for approval by the Research Ethics Committee.

### Variables and measurement

Data tabulation based on this website consisted of inserting the types of violence in the row (one at a time), and the variable “year of notification” in the column. The variable “sex” was used as a selection filter to collect data relating to female victims. The types of violence analyzed were: Self-harm, physical, psychological or moral violence, torture, sexual violence, human trafficking, financial or economic violence, violence through neglect or abandonment, child labor, legal intervention and other types of violence. Violence with fewer than 10,000 reported cases (<0.5%) were grouped into the category of other violence (human trafficking, child labor, and legal intervention). 

The following variables were used to analyze the epidemiological profile of the notifications: age group (≤9 years | 10-19 years | 20-59 years | ≥60 years), education (up to 4th incomplete grade of elementary school | 4th-8th incomplete grade of elementary school | Complete elementary school to incomplete high school | Complete high school to incomplete higher education | Complete higher education), race/skin color (White | Black | Yellow | Brown | Indigenous), geographic region (North | Northeast | Southeast | South | Center-West), means of aggression (Bodily force/beating | Choking | Blunt object | Sharp object | Hot substance/object | Poisoning/intoxication | Firearm | Threat | Others), kinship relationship between perpetrator and victim (Father | Mother | Stepfather | Stepmother | Spouse | Ex-spouse | Boyfriend/girlfriend | Ex-boyfriend/girlfriend | Son/daughter | Brother/sister | Friend/acquaintance | Stranger | Caregiver | Employer/boss | Person with institutional relationship | Police officer/law enforcement officer | Self | Others), place of occurrence (Residence | Collective housing | School | Sports venue | Bar or similar | Public road | Commerce/services | Industries/construction | Others), referral to health sector (Outpatient | Hospital admission) and evolution of the case (Discharge | Escape/flight | Death by violence | Death by other causes). All response categories were maintained as presented by SINAN, with the exception of age group and education level, where some categories were grouped together. This clustering was based on national health surveys such as the Brazilian National Health Survey (12) and the National School Health Survey (13), both conducted by the Brazilian Institute of Geography and Statistics.

All variables were organized in a single database in Excel, where the internal consistencies of the information were analyzed and, later, used for statistical analyses in the Stata statistical software (version 14.2). 

### Statistical methods

The temporal trend of notifications of violence against women was calculated using Prais-Winsten linear regression, which allowed notification trends to be analyzed as stable (p-value>0.05), decreasing (p-value<0.05 and negative regression coefficient) or increasing (p-value<0.05 and positive regression coefficient) (14). This coefficient represents the annual variation, either in the total period from 2014 to 2023, or in the most recent period from 2020 to 2023.

The dependent variables were composed of two indicators: i) Incidence rate for each type of violence (absolute number of notifications of each type of violence, divided by the total resident female population, multiplied by 100 thousand), and ii) Proportion of each type of violence (absolute number of notifications of each type of violence, divided by the sum of notifications of violence among women, multiplied by 100). These indicators were calculated for each year of interest, and the year was the independent variable in both models. 

Sociodemographic characteristics (age group, education, race/skin color, geographic region) and characteristics of the incident (means of aggression, kinship between perpetrator and victim, location of incident, referral to the health sector and case evolution) were analyzed using absolute and relative frequencies. 

## Results

Reports of violence against women have shown an increasing trend over the last 10 years, with an annual variation of 9.8 cases per 100,000 women (p-value<0.05). This trend was observed for all types of violence, with emphasis on self-harm (16.4 cases per 100,000 women per year), financial/economic violence (11.9 cases per 100,000 women per year) and sexual violence (10.8 cases per 100,000 women per year). Recently, the magnitude of the increase in violence against women has almost doubled to 19.2 cases per 100,000 women per year (p-value<0.05). This scenario was observed for almost all types of violence (Table 1). 

**Table 1:** Annual variation in the incidence rate (per 100,000 women) for notifications of violence against women. Brazil, 2014–2023 (n=3.861.994)

Types of violence	Year										Annual variation (Percentage/year) (2014/2023)	Annual variation (Percentage/year) (2020/2023)
	2014	2015	2016	2017	2018	2019	2020	2021	2022	2023		
Self-harm	19.3	25.1	28.8	44.0	57.8	84.1	62.0	74.7	89.6	117.3	16.4^a^	20.6^a^
Physical violence	94.9	101.5	110.0	133.6	140.9	152.2	120.0	123.3	142.3	192.2	6.2^a^	16.0
Psychological/moral violence	45.0	50.3	52.2	59.9	59.0	64.4	53.9	57.5	67.5	91.4	6.5^a^	17.8^a^
Violence torture	4.1	4.7	5.0	6.4	5.5	5.9	5.0	5.1	5.9	7.5	4.5^a^	14.0^a^
Sexual violence	23.6	23.5	26.1	31.3	34.6	37.7	32.5	38.6	46.4	63.3	10.8^a^	21.6^a^
Financial/economic violence	2.4	2.6	3.0	3.2	3.7	4.5	3.7	4.4	5.3	7.6	11.9^a^	23.5^a^
Violence neglect/abandonment	12.9	14.1	15.7	19.7	20.6	21.2	16.7	20.3	23.1	32.3	8.6^a^	15.2
Other violence	11.7	15.2	17.6	28.0	42.7	58.0	45.7	51.0	65.1	91.1	18.6^a^	23.7^a^
Total	214.0	237.0	258.4	326.0	364.7	427.9	339.6	374.9	445.3	602.9	9.8^a^	19.2^a^

^a^Statistically significant annual variation (p-value<0.05).

More than 220,000 notifications of violence against women were made to the National System of Injuries and Notifications (SINAN) for interpersonal and self-inflicted violence in 2014, and this number increased by around 3 times in 10 years, with almost 670,000 notifications made in 2023 (Table 2). Of the total number of notifications, physical and psychological/moral violence stood out throughout the period analyzed, with an average proportion of 37.7% and 17.4%, respectively. An increase of 8.1% per year was observed (p-value<0.05) in the proportion of notifications for self-inflicted injury, while most other types of violence showed a reduction in their proportion, with emphasis on torture, sexual violence, and psychological/moral violence (-5.6%; -4.1% and -4.0%/year, respectively). In the most recent period (2020-2023), only cases of torture continued to reduce (-4.9%/year) (Table 2).

**Table 2:** Annual variation in the proportion (%) of notifications of violence against women. Brazil, 2014–2023 (n=3.861.994)

Types of violence	Year										Annual variation (%) (2014/2023)	Annual variation (%) (2020/2023)
2014	2015	2016	2017	2018	2019	2020	2021	2022	2023
Self-harm	9.0	10.6	11.2	13.5	15.8	19.7	18.3	19.9	20.1	19.5	8,1^a^	1.9
Physical violence	44.3	42.8	42.6	41.0	38.6	35.6	35.3	32.9	32.0	31.9	-4.1^a^	-3.4
Psychological/moral violence	21.0	21.2	20.2	18.4	16.2	15.0	15.9	15.3	15.2	15.2	-4.0^a^	-1.5
Violence torture	1.9	2.0	1.9	2.0	1.5	1.4	1.5	1.4	1.3	1,2	-5.6^a^	-4.9^a^
Sexual violence	11.0	9.9	10.1	9.6	9.5	8.8	9.6	10.3	10.4	10.5	-0.3	2.7
Financial/economic violence	1.1	1.1	1,2	1.0	1.0	1.1	1.1	1,2	1,2	1.3	1.3	4.5^a^
Violence neglect/abandonment	6.0	6.0	6.1	6.0	5.6	5.0	4.9	5.4	5.2	5.4	-1.8	1.3
Other violence	5.5	6.4	6.8	8.6	11.7	13.5	13.5	13.6	14.6	15.1	10.3^a^	4.6^a^
Total	220,324	246,201	270,747	344,397	388,486	459,495	367,574	408,850	489,123	666,797	-	-

^a^Statistically significant annual variation (p-value<0.05).

In general, the main sociodemographic characteristics related to the notifications were: age range of 20-59 years, 4th to 8th incomplete grade of elementary school, brown race/skin color and from the Southeast region. The different sociodemographic profile for some types of violence stands out, such as: self-harm (women of white race/skin color), sexual violence (women aged 10-19) and violence due to neglect/abandonment (women aged 9 or younger). Although 4th to 8th incomplete grade education prevailed in the total number of notifications, it is worth noting that education from complete secondary education to incomplete higher education stood out for most of the types of violence analyzed, with the exception of sexual violence and neglect/abandonment (Table 3). 

**Table 3:** Absolute and relative frequency of notifications of violence against women according to sociodemographic characteristics. Brazil, 2014–2023 (n=3.861.994)

Sociodemographic characteristics	Self-harm	Physical violence	Psychological/moral violence	Torture	Sexual violence	Financial/economic violence	Neglect/abandonment
	n (%)	n (%)	n (%)	n (%)	n (%)	n (%)	n (%)
**Age group** (years)							
≤9	5,810 (0.9)	58,375 (4.2)	41,427 (6.4)	3,724 (6.3)	102,764 (26.7)	1,407 (3.2)	115,887 (54.8)
10-19	211,003 (32.4)	262,655 (18.7)	116,558 (18.1)	11,747 (19.9)	177,278 (46.1)	2,879 (6.6)	42,737 (20.2)
20-59	416,503 (63.9)	1,022,481 (72.7)	447,794 (69.5)	40,837 (69.1)	100,275 (26.1)	30,546 (70.4)	13,724 (6.5)
≥60	16,829 (2.6)	60,866 (4.3)	38,090 (5.9)	2,648 (4.5)	4,059 (1.1)	8,468 (19.5)	38,517 (18.2)
Ignored/blank	1,161 (0.2)	1,880 (0.1)	870 (0.1)	100 (0.2)	396 (0.1)	62 (0.1)	526 (0.2)
Education							
Up to 4th grade of incomplete elementary school	21,439 (3.3)	84,594 (6.0)	49,722 (7.7)	5,307 (9.0)	34,879 (9.1)	4,936 (11.4)	20,922 (9.9)
4th to 8th grade of incomplete elementary school	98,953 (15.2)	222,085 (15.8)	117,181 (18.2)	11,387 (19.3)	102,389 (26.6)	6,904 (15.9)	23,483 (11.1)
Completed Elementary Education to Incomplete High School Education	126,255 (19.4)	239,207 (17.0)	111,367 (17.3)	10,564 (17.9)	51,467 (13.4)	6,201 (14.3)	10,858 (5.1)
Completed secondary education to incomplete higher education	139,759 (21.5)	280,779 (20.0)	144,638 (22.4)	11,553 (19.6)	43,217 (11.2)	10,606 (24.5)	4,244 (2.0)
Completed Higher Education	20,397 (3.1)	41,297 (2.9)	30,240 (4.7)	1,987 (3.4)	8,704 (2.3)	2,932 (6.8)	722 (0.3)
Ignored/blank	244,503 (37.5)	538,295 (38.3)	191,591 (29.7)	18,258 (30.9)	144,116 (37.5)	11,783 (27.2)	151,162 (71.5)
**Race/skin color**							
White	301,742 (46.3)	533,052 (37.9)	252,644 (39.2)	20,966 (35.5)	135,028 (35.1)	16,143 (37.2)	82,174 (38.9)
Black	41,170 (6.3)	129,790 (9.2)	66,547 (10.3)	6,202 (10.5)	36,047 (9.4)	5,707 (13.2)	13,608 (6.4)
Yellow (Asian)	5,688 (0.9)	11,388 (0.8)	5,349 (0.8)	582 (1.0)	3,191 (0.8)	462 (1.1)	1,214 (0.6)
Brown	244,570 (37.6)	588,119 (41.8)	273,570 (42.4)	27,633 (46.8)	180,189 (46.8)	18,595 (42.9)	89,659 (42.4)
Indigenous	3.175 (0.5)	13,120 (0.9)	5,431 (0.8)	633 (1.1)	4,788 (1.2)	348 (0.8)	1,673 (0.8)
Ignored/blank	54,961 (8.4)	130,788 (9.3)	41,198 (6.4)	3,040 (5.1)	25,529 (6.6)	2,107 (4.9)	23,063 (10.9)
**Geographic region**							
North	25,708 (3.9)	81,087 (5.8)	55,716 (8.6)	5,534 (9.4)	55,065 (14.3)	2,702 (6.2)	9,661 (4.6)
Northeast	101,621 (15.6)	231,662 (16.5)	115,151 (17.9)	12,857 (21.8)	71,032 (18.5)	13,909 (32.1)	42,704 (20.2)
Southeast	314,387 (48.3)	785,711 (55.9)	321,805 (49.9)	27,418 (46.4)	153,739 (40.0)	16,759 (38.6)	73,130 (34.6)
South	148,516 (22.8)	218,968 (15.6)	115,776 (18.0)	8,343 (14.1)	69,001 (17.9)	7,881 (18.2)	65,370 (30.9)
Center-West	61,026 (9.4)	88,625 (6.3)	36,223 (5.6)	4,889 (8.3)	35,849 (9.3)	2,105 (4.9)	20,492 (9.7)
Ignored/blank	48 (0.0)	204 (0.0)	68 (0.0)	15 (0,0)	86 (0.0)	6 (0.6)	34 (0.0)
Total	651,306 (100.0)	1,406,257 (100.0)	644,739 (100.0)	59,056 (100.0)	384,772 (100.0)	43,362 (100.0)	211,391 (100.0)

The characteristics of the occurrence can be seen in tables 4 and 5. The most commonly used means of aggression in cases of interpersonal violence were physical force/beating and threats, while in cases of self-harm, poisoning/intoxication (61.4%) were used. Regarding the relationship between the perpetrator of violence and the victim, the spouse stands out in cases of torture (29.6%), financial/economic violence (29.2%), psychological/moral violence (27.6%) and physical violence (26.2%); the mother in cases of violence due to neglect/abandonment (45.9%); and a friend/acquaintance in cases of sexual violence (23.1%); and the person themselves in cases of self-harm (94.9%) (Table 4). This proportion below 100% alerts us to the possibility of overlapping types of violence in the notification forms, even though the Ministry of Health’s instructions advise only indicating the main type of violence for purposes of registration in the National System of Injuries and Notifications (SINAN) for interpersonal and self-inflicted violence (6). 

**Table 4:** Absolute and relative frequency of notifications of violence against women according to characteristics of the incidents. Brazil, 2014–2023 (n=3.861.994)

Characteristics of the incident	Self-harm	Physical violence	Psychological/moral violence	Torture	Sexual violence	Financial/economic violence	Neglect/abandonment
	n (%)	n (%)	n (%)	n (%)	n (%)	n (%)	n (%)
**Means of aggression**							
Bodily force/beating	22,001 (3.2)	1,006,287 (54.4)	384,839 (42.1)	47,182 (38.9)	146,357 (41.7)	23,889 (33.4)	14,053 (9.6)
Choking	25,996 (3.8)	100,122 (5.4)	55,481 (6.1)	14,925 (12.3)	12,503 (3.6)	5,332 (7.5)	1,446 (1.0)
Blunt object	9,660 (1.4)	88,422 (4.8)	35,006 (3.8)	7,501 (6.2)	6,504 (1.9)	3,350 (4.7)	2,786 (1.9)
Sharp object	109,183 (15.8)	164,026 (8.9)	46,356 (5.1)	10,756 (8.9)	12,759 (3.6)	3,546 (5.0)	2,805 (1.9)
Hot substance/object	6,708 (1.0)	11,791 (0.6)	4.108 (0.4)	1,426 (1.2)	1,687 (0.5)	510 (0.7)	6,157 (4.2)
Poisoning/intoxication	423,355 (61.4)	114,987 (6.2)	19,027 (2.1)	1,428 (1.2)	5,257 (1.5)	523 (0.7)	12,355 (8.5)
Firearm	1,671 (0.2)	31,544 (1.7)	12,987 (1.4)	3,232 (2.7)	10,543 (3.0)	1,302 (1.8)	626 (0.4)
Threat	5,732 (0.8)	252,342 (13.6)	295,708 (32.4)	31,030 (25.6)	98,028 (27.9)	27,174 (38.0)	10,738 (7.3)
Other	84,733 (12.3)	79,803 (4.3)	59,957 (6.6)	3,831 (3.2)	57,156 (16.3)	5,804 (8.1)	95,131 (65.1)
**Family relationship between perpetrator and victim**							
Father	2,587 (0.4)	42,637 (3.1)	33,170 (5.0)	2,583 (4.1)	33,912 (8.8)	1,608 (3.4)	68,667 (24.6)
Mother	4,270 (0.7)	42,215 (3.1)	26,345 (3.9)	2,300 (3.7)	7,008 (1.8)	1,852 (3.9)	128,012 (45.9)
Stepfather	580 (0.1)	20,181 (1.5)	18,310 (2.7)	1,852 (3.0)	35,963 (9.4)	484 (1.0)	4,116 (1.5)
Stepmother	167 (0.0)	3,696 (0.3)	2,170 (0.3)	268 (0.4)	727 (0.2)	115 (0.2)	905 (0.3)
Spouse	6,877 (1.1)	355,236 (26.2)	184,245 (27.6)	18,549 (29.6)	20,297 (5.3)	13,976 (29.2)	5,390 (1.9)
Ex-spouse	1,979 (0.3)	131,883 (9.7)	97,182 (14.5)	7,160 (11.4)	10,029 (2.6)	8,206 (17.2)	1,302 (0.5)
Boyfriend/girlfriend	1,690 (0.3)	62,135 (4.6)	27,175 (4.1)	3.125 (5.0)	27,472 (7.2)	1,467 (3.1)	2,081 (0.7)
Ex-boyfriend/girlfriend	696 (0.1)	38,556 (2.8)	25,198 (3.8)	2,113 (3.4)	6,843 (1.8)	1,753 (3.7)	572 (0.2)
Son(daughter)	1,383 (0.2)	41,978 (3.1)	28,496 (4.3)	1,847 (2.9)	875 (0.2)	6,366 (13.3)	25,380 (9.1)
Brother(sister)	1,299 (0.2)	45,480 (3.4)	20,432 (3.1)	1,389 (2.2)	8,413 (2.2)	1,448 (3.0)	4,536 (1.6)
Friend/acquaintance	3,723 (0.6)	146,703 (10.8)	64,733 (9.7)	5,813 (9.3)	88,648 (23.1)	1,975 (4.1)	4,771 (1.7)
Stranger	2.101 (0.3)	119,447 (8.8)	42,203 (6.3)	8,022 (12.8)	77,095 (20.1)	2,151 (4.5)	2,512 (0.9)
Caregiver	196 (0.0)	2,932 (0.2)	2,162 (0.3)	300 (0.5)	2,588 (0.7)	540 (1.1)	3,995 (1.4)
Employer/boss	134 (0.0)	2,001 (0.1)	2,984 (0.4)	178 (0.3)	1,577 (0.4)	279 (0.6)	173 (0.1)
Person with institutional relationship	657 (0.1)	6,816 (0.5)	5,011 (0.7)	310 (0.5)	3,772 (1.0)	168 (0.4)	1,450 (0.5)
Police Officer/Law Enforcement Officer	463 (0.1)	5.113 (0.4)	1,946 (0.3)	376 (0.6)	681 (0.2)	155 (0.3)	169 (0.1)
Own person	620,663 (94.9)	193,445 (14.3)	29,073 (4.3)	2,731 (4.4)	1,512 (0.4)	585 (1.2)	9,632 (3.5)
Other	4,712 (0.7)	95,706 (7.1)	57,601 (8.6)	3,761 (6.0)	56,458 (14.7)	4,685 (9.8)	15,303 (5.5)

Home was the most reported place of occurrence for all types of violence, ranging from 63.3% for sexual violence to 85.7% for self-inflicted injury. Regarding referral to the health sector and case evolution, more than 90.0% of notifications did not receive valid responses (data ignored, blank or not applicable). Among the valid data, the outpatient sector and discharge stand out (Table 5).

**Table 5:** Absolute and relative frequency of notifications of violence against women according to characteristics of the incidents. Brazil, 2014–2023 (n=3.861.994)

Characteristics of the incident	Self-harm	Physical violence	Psychological/moral violence	Torture	Sexual violence	Financial/economic violence	Neglect/abandonment
	n (%)	n (%)	n (%)	n (%)	n (%)	n (%)	n (%)
**Place of occurrence**							
At home	558,159 (85.7)	931,295 (66.2)	478,196 (74.2)	42,481 (71.9)	243,382 (63.3)	37,087 (85.5)	141,009 (66.7)
Collective housing	3,685 (0.6)	8,588 (0.6)	5,138 (0.8)	506 (0.9)	3,087 (0.8)	266 (0.6)	1,303 (0.6)
School	7,041 (1.1)	19,201 (1.4)	8,726 (1.4)	530 (0.9)	7,870 (2.0)	106 (0.2)	2,140 (1.0)
Sports venue	429 (0.1)	3,067 (0.2)	1,373 (0.2)	153 (0.3)	1,234 (0.3)	28 (0.1)	312 (0.1)
Bar or similar	1,688 (0.3)	31,865 (2.3)	9,228 (1.4)	979 (1.7)	4,918 (1.3)	305 (0.7)	691 (0.3)
Public road	21,577 (3.3)	192,572 (13.7)	67,107 (10.4)	7,931 (13.4)	41,717 (10.8)	2,518 (5.8)	11,542 (5.5)
Commercial/services building	3,147 (0.5)	17,319 (1.2)	10,770 (1.7)	556 (0.9)	4,873 (1.3)	632 (1.5)	6,843 (3.2)
Plant/construction	257 (0.0)	1,276 (0.1)	752 (0.1)	126 (0.2)	902 (0.2)	42 (0.1)	66 (0.0)
Other	12,814 (2.0)	52,153 (3.7)	32,930 (5.1)	3,521 (6.0)	37,566 (9.8)	1,349 (3.1)	25,573 (12.1)
Ignored/blank	42,509 (6.5)	148,921 (10.6)	30,519 (4.7)	2,273 (3.8)	39,223 (10.2)	1,029 (2.4)	21,912 (10.4)
**Health sector referrals**							
Outpatient	11,771 (1.8)	42,907 (3.1)	18,365 (2.8)	2.008 (3.4)	13,680 (3.6)	811 (1.9)	4.186 (2.0)
Hospital admission	4,246 (0.7)	10,079 (0.7)	2,874 (0.4)	451 (0.8)	2,064 (0.5)	194 (0.4)	2,372 (1.1)
Ignored/blank/not applicable	635,289 (97.5)	1,353,271 (96.2)	623,500 (96.7)	56,597 (95.8)	369,028 (95.9)	42,357 (97.7)	204,833 (96.9)
**Case evolution**							
Discharge	13,395 (2.1)	64,688 (4.6)	28,191 (4.4)	2,628 (4.5)	13,920 (3.6)	1,397 (3.2)	6,098 (2.9)
Evasion/escape	361 (0.1)	981 (0.1)	376 (0.1)	41 (0.1)	195 (0.1)	30 (0.1)	937 (0.4)
Death by violence	334 (0.1)	764 (0.1)	81 (0.0)	49 (0.1)	28 (0.0)	2 (0,0)	51 (0.0)
Death from other causes	25 (0.0)	48 (0.0)	21 (0.0)	6 (0.0)	7 (0.0)	7 (0.0)	63 (0.0)
Ignored/blank	637,191 (97.8)	1,339,776 (95.3)	616,070 (95.6)	56,332 (95.4)	370,622 (96.3)	41,926 (96.7)	204,242 (96.6)

## Discussion

This study showed that notifications of violence against women have shown an increasing trend in the last decade, especially after the COVID-19 pandemic. Among the types of violence, the increase in reports of self-inflicted injuries, sexual and financial/economic violence stands out. However, proportionally, the following stand out: i) physical and psychological/moral violence as the main types of violence against women in Brazil, and ii) an increase in cases of self-harm compared to cases of interpersonal aggression. Reports of adult women (20-59 years old) with 4th to 8th incomplete grade of elementary school education and of brown race/skin color prevailed. Among the cases of interpersonal violence, physical force/beating and threats stood out as means of aggression, and among the cases of self-harm, poisoning/intoxication. The spouse was the main aggressor, and the home environment was the main place of occurrence. 

The increase in these notifications may be a reflection of two scenarios: i) An effective increase in violence against women due to the historical, cultural and social construction of a sexist society, which continues to this day as the main obstacle to female empowerment and effective gender equality (15,16); and ii) An increase in the recording of notifications of violence in Health Information Systems, whether due to technical and operational improvements (including staff training and computerization of Units) or advances in the support network for women (social and family networks, civil society organizations, public facilities). The 2011 National Policy to Combat Violence against Women boosted these advances by establishing a support network that offers services related to health (such as Women’s Reference Centers), social assistance (such as Shelters), justice (such as Women’s Centers in Public Defender’s Offices and Domestic Violence Courts) and security (such as Specialized Police Stations) (3,4,17).

This increase in notifications almost doubled in the recent period of analysis, consistent with national and international studies that point to the COVID-19 pandemic as a possible justification (18-21). On the one hand, given the restrictions on social interaction, there was an increase in coexistence between the victim and the potential aggressor, intensifying the level of stress, the consumption of alcoholic beverages and other drugs, uncertainty about the future and the reduction of family income. On the other hand, although the increase in violence was detected in the results of the present study, underreporting must have been intensified, considering the disarticulation of the support and protection network for these women during the pandemic period, with limited access to the healthcare network, and consequently, discouraging women from effectively reporting violence (22,23). 

Among the types of violence, a proportional increase in reports of self-inflicted injuries was observed to the detriment of cases of interpersonal violence between 2014 and 2023. Violence tends to trigger and intensify the illness process in victims, whether physical and/or psycho-emotional, acute and/or chronic, short and/or long-term, subjected to prolonged drug treatments and/or therapies (24). This illness sometimes intensifies the cycle of violence, potentially increasing suicide and self-harm rates (25). In Brazil, self-harm mainly affects women, with suicide being more prevalent among younger women (15-19 years old) (26).

Additionally, analyzing the sociodemographic profile of victims not only allows for the identification of the groups most vulnerable to violence, but also helps us to contextualize the profile of women with greater access to the care network, and therefore those with most notifications. Adult women (20-59 years old), with 4th to 8th incomplete grade of elementary school, of brown race/skin color and from the Southeast region were the predominant profile for reporting violence, however, it cannot be said that this is the profile of the main victims in Brazil. The North and Northeast, for example, tend to be the geographic regions with the greatest underreporting of these cases (27). Furthermore, it is known that the sociodemographic profile of victims varies depending on the type of violence, but in general, the greater the vulnerability of the woman, the greater the chance of suffering violence, and often, not reporting it, due to lack of knowledge or fear (22). In the present study, the greatest vulnerability was observed for notifications of sexual violence and neglect/abandonment. 

The most commonly used means of aggression were physical force/beating and assault in cases of interpersonal violence, and poisoning/intoxication in cases of self-harm. It is worth noting that physical force/beating has been used less and less in some types of violence, such as sexual violence (8), while threats have been an increasingly used means of aggression, especially when the victims are younger (10-19 years old), as was the case of sexual violence in the present study. In addition to sexual violence, one of the highest proportions of threats was identified in financial/economic violence and psychological/moral violence, where the spouse was the main perpetrator and the residence was the main place of occurrence. Domestic violence usually follows a well-defined cycle, with threats and physical aggression coexisting. Initially, there are disagreements and provocations, followed by threatening strategies such as separation and preventing participation in the children’s lives. And, finally, the physical aggression happens (28).

Women who are victims of violence need to be monitored by a multidisciplinary team and surrounded by an effective support network, with the aim of not only reducing the incidence of interpersonal violence, but also improving the quality of life of these women and reducing the chance of self-harm. However, more than 90.0% of notifications did not contain valid information about the referral of the case in the health sector and its clinical evolution. This absence or failures in filling out notification forms may be a reflection of the fact that healthcare professionals still see the notification as a complaint, making epidemiological analysis of the problem difficult (29). Health care units are required to refer patients only in special situations, such as child or adolescent victims (Tutelary Council or Public Prosecutor’s Office) and elderly victims (Municipal Council for the Elderly or Public Prosecutor’s Office). In general, health teams are instructed to inform about the existence of social protection network services and the importance of reporting, but they should not forward the case without explicit authorization (30).

Contextualizing the notifications allows us to analyze the profile of violence to which women are subjected, but also to make a critical analysis of the country’s notification process. These systems must not only provide quality information, but also support practical improvements for the population (5). In that regard, potential limitations should be noted when assessing the results of this study: i) underreporting of cases of violence in Brazil; ii) partial completion of notification forms; and iii) limitations in identifying cases with overlapping violence (involving more than one type). This overlap of types of violence limited the interpretation of some results of the manuscript, such as means of aggression and kinship relationship between perpetrator and victim. However, it is believed that these limitations do not outweigh the potential of the study. The data comes from a Health Information System regulated and consolidated throughout the national territory (6,7), providing a basis for discussions to improve Information Systems and the Unified Health System. In order to reduce the limitation of underreporting in the system, the sum of occurrences by type of violence was established as the study subjects. This number was greater than the total number of notifications actually made (3,861,994 versus 2,503,337). 

Finally, we concluded that all types of violence against women (self-harm, physical, psychological/moral violence, torture, sexual, financial/economic violence, neglect/abandonment, and others) showed an increase in notification in the country between 2014 and 2023, with an intensification of this magnitude in the last four years (post-COVID-19 pandemic). This shows that Brazil is on the wrong track to achieving the goal of “eliminating all forms of violence against all women and girls in the public and private spheres worldwide by 2030”, as agreed with the United Nations through Sustainable Development Goal 5 (Gender Equality) (31). Women aged 20-59, with 4th to 8th incomplete grade of elementary school, brown race/skin color and from the Southeast region prevailed among notifications of violence. However, this profile was different depending on the type of violence, with greater vulnerability being highlighted among those who suffered sexual violence and neglect/abandonment. Bodily force/beating and threats were highlighted as the main means of aggression, the victim’s spouse as the main perpetrator of violence, and the victim’s home as the main place of occurrence. More than 90.0% of the information regarding the referral of the case to the health sector and the evolution of the case were not valid. 

## Data Availability

The database and analysis codes used in this study are available at https://datasus.saude.gov.br/.
