# Supplementary material for: Temporal trend and epidemiological profile of notifications of violence against women in Brazil: 2014-2023
Source: Epidemiol Serv Saude. 2025 Aug 8;34:e20240475. doi: 10.1590/S2237-96222025v34e20240475.en (PMC12342726; doi:10.1590/S2237-96222025v34e20240475.en)
Supplement: Translation

# Translation: Tendência temporal e perfil epidemiológico das notificações de violência contra as mulheres no Brasil: 2014-2023

**DOI:** 10.1590/S2237-96222025v34e20240475.pt

**Authors:** Geovanna Carvalho Cardoso Lima, Camila Mendes dos Passos, Alba Lúcia Santos Pinheiro, Ícaro José Santos Ribeiro, Emanuella Gomes Maia

**Affiliations:** 1 Universidade Estadual de Santa Cruz, Programa de Pós-Graduação em Enfermagem e Saúde da Universidade Federal da Bahia, Salvador, Bahia, Brasil; 2 Universidade Federal de Viçosa, Departamento de Medicina e Enfermagem, Viçosa, Minas Gerais, Brasil; 3 Universidade Estadual de Santa Cruz, Departamento de Ciências da Saúde, Ilhéus, Bahia, Brasil; 4 Universidade Estadual de Santa Cruz, Programa de Pós-Graduação em Enfermagem, Ilhéus, Bahia, Brasil

**Keywords:** Violência, Mulheres, Sistemas de Informação em Saúde, Fatores de Tempo, Saúde Pública

## Abstract

**Objetivo:**

Analisar a tendência temporal e o perfil epidemiológico das notificações de violência contra as mulheres no Brasil entre 2014 e 2023.

**Métodos:**

Trata-se de uma série temporal, realizada a partir do Sistema de Informação de Agravos de Notificação de Violência Interpessoal e Autoprovocada. Estatística descritiva e regressão de Prais-Winsten foram utilizadas para análise dos dados.

**Resultados:**

As notificações de violência contra as mulheres apresentaram tendência crescente entre 2014 e 2023, principalmente após o ano de 2020 (de 9,8 para 19,2 casos por 100 mil mulheres ao ano). Proporcionalmente, destacou-se as lesões autoprovocadas em detrimento das agressões interpessoais. A maioria das notificações referiram às mulheres adultas (54,0%), da 4ª até 8ª série do Ensino Fundamental incompleto (17,4%) e de raça/cor da pele parda (43,0%). A força corporal/espancamento prevaleceu como meio de agressão (31,9%), realizada pelo cônjuge (17,3%) e em ambiente domiciliar (73,4%).

**Conclusão:**

A violência contra a mulher aumentou na última década, com intensificação desse aumento nos últimos anos. As mulheres com vulnerabilidade social foram mais atingidas, principalmente nos casos de violência sexual e de negligência/abandono.

**Aspectos éticos**

Esta pesquisa utilizou bancos de dados de domínio público e anonimizados.

## Introdução

Os papéis sociais estabelecidos desde a antiguidade, como a submissão do sexo feminino, refletem a complexidade cultural, econômica e política que envolve a violência contra as mulheres no mundo e no Brasil. Segundo a Organização Mundial de Saúde, em 2018, três a cada dez mulheres com idade igual ou maior que 15 anos foram submetidas pelo menos uma vez a episódios de violência física e/ou sexual, por agressores do sexo masculino, independente de possuir vínculo afetivo com a vítima (1). No Brasil, por sua vez, segundo dados do Atlas da violência de 2022, mais de 50 mil assassinatos de mulheres foram registrados no país entre 2009 e 2019, sendo as principais vítimas, as mulheres negras (2). 

A violência contra as mulheres é um grave problema de saúde pública e uma violação dos direitos humanos a qual essas mulheres são submetidas diariamente. A Convenção de Belém do Pará de 1994 definiu a violência contra as mulheres como qualquer conduta ou ação fundamentada no gênero, que cause sofrimento sexual, psicológico ou físico, dano ou morte à mulher, no âmbito privado e público (3). Essa definição abrange desde a violência autoprovocada/autoinfligida (contra si mesmo), até as violências interpessoais, como violência doméstica/intrafamiliar (que ocorre entre os parceiros íntimos e entre os membros da família, principalmente no ambiente residencial, mas não exclusivamente) e a violência extrafamiliar/comunitária (que ocorre no ambiente social, em geral, entre conhecidos ou desconhecidos) (4).

Nesse contexto, os sistemas de vigilância do país são evidenciados como instrumentos importantes para o reconhecimento do perfil de saúde, exposições e condições de risco da população (5). O sistema de vigilância de violências e acidentes é composto por dois componentes: Sistema de Informação de Agravos de Notificação de Violência Interpessoal e Autoprovocada e Vigilância de Violências e Acidentes em Serviços Sentinelas de Urgência e Emergência. O Sistema de Informação de Agravos de Notificação de Violência Interpessoal e Autoprovocada, de interesse da presente pesquisa, compõe a vigilância contínua do país por meio da notificação compulsória de violência interpessoal e autoprovocada. A notificação compulsória caracteriza-se como a comunicação obrigatória à autoridade de saúde sobre a ocorrência de suspeita ou confirmação de doença, agravo ou evento de saúde pública (4). Em 2003, a Lei nº 10.778 foi publicada para estabelecer essa notificação em território nacional (6), e em 2004, a Portaria nº 2.406 aprovou os respectivos instrumentos, protocolos e fluxos (7). 

Ainda que os dados do Sistema de Informação de Agravos de Notificação de Violência Interpessoal e Autoprovocada tenham subsidiado a realização de alguns estudos, boa parte limitou-se a analisar um tipo de violência (8,9), ou área específica do país (10,11). Nesse sentido, com o intuito de contribuir com o cenário de evidências sobre a violência contra as mulheres, o objetivo desse trabalho foi analisar a tendência temporal e o perfil epidemiológico das notificações de violência contra as mulheres no Brasil entre 2014 e 2023. Acredita-se que essas notificações tenham aumentado na última década, principalmente após a pandemia da COVID-19, e que tenham atingido principalmente as mulheres mais vulneráveis socioeconomicamente. 

## Métodos

### Delineamento

Trata-se de uma série temporal, retrospectiva, de abordagem quantitativa, norteada pelo checklist *Strengthening the Reporting of Observational Studies in Epidemiology*. 

### Participantes e fonte de dados

A população em estudo foi formada pela somatória de notificações de violência contra o sexo feminino no Brasil no período de 2014 a 2023. O banco de dados para a realização desta pesquisa foi o Sistema de Informação de Agravos de Notificação de Violência Interpessoal e Autoprovocada (SINAN). Esse sistema é alimentado por informações presentes na ficha de notificação, que por sua vez, deve ser preenchida e registrada por todos os órgãos públicos de saúde, entidades conveniadas e contratadas, mediante qualquer caso suspeito ou confirmado de violência contra as mulheres, independentemente da idade da vítima (4).

A coleta de dados foi realizada entre novembro e dezembro de 2024 no site do Departamento de Informática do Sistema Único de Saúde, do Ministério da Saúde do Brasil, por meio do link <https://datasus.saude.gov.br/>. Trata-se de dados secundários de acesso público, com garantia do sigilo e anonimato de todas as participantes cujas notificações foram realizadas. O estudo está em consonância com as recomendações da Resolução do Conselho Nacional de Saúde nº 466/2012, com dispensa a aprovação do Comitê de Ética em Pesquisa.

### Variáveis e mensuração

Ao acessar esse site, a tabulação dos dados consistiu em inserir os tipos de violência na linha (uma de cada vez), e a variável “ano de notificação” na coluna. A variável “sexo” foi utilizada como filtro de seleção para coletar os dados referentes às vítimas do sexo feminino. Os tipos de violência analisados foram: Lesão autoprovocada, violência física, psicológica ou moral, tortura, violência sexual, tráfico de seres humanos, violência financeira ou econômica, violência por negligência ou abandono, trabalho infantil, intervenção legal e outros tipos de violência. As violências com notificação menor do que 10 mil casos (<0,5%) foram agrupadas à categoria de outras violências (tráfico de seres humanos, trabalho infantil, e intervenção legal). 

As seguintes variáveis foram utilizadas para analisar o perfil epidemiológico das notificações: faixa etária (≤9 anos | 10-19 anos | 20-59 anos | ≥60 anos), escolaridade (até 4ª série do ensino fundamental incompleta | 4ª-8ª série do ensino fundamental incompleta | Ensino fundamental completo até ensino médio incompleto | Ensino médio completo até ensino superior incompleto | Ensino superior completo), raça/cor da pele (Branca | Preta | Amarela | Parda | Indígena), região geográfica (Norte | Nordeste | Sudeste | Sul | Centro-Oeste), meios de agressão (Força corporal/espancamento | Enforcamento | Objeto contundente | Objeto perfuro cortante | Substância/objeto quente | Envenenamento/intoxicação | Arma de fogo | Ameaça | Outros), relação de parentesco entre autor e vítima (Pai | Mãe | Padrasto | Madrasta | Cônjuge | Ex-cônjuge | Namorado(a) | Ex-namorado(a) | Filho(a) | Irmão(ã) | Amigo(a)/conhecido(a) | Desconhecido(a) | Cuidador(a) | Patrão/chefe | Pessoa com relação institucional | Policial/agente da lei | Própria pessoa | Outros), local de ocorrência (Residência | Habitação coletiva | Escola | Local de prática esportiva | Bar ou similar | Via pública | Comércio/serviços | Indústrias/construção | Outros), encaminhamento no setor saúde (Ambulatorial | Internação hospitalar) e evolução do caso (Alta | Evasão/fuga | Óbito por violência | Óbito por outras causas). Todas as categorias de respostas foram mantidas conforme apresentadas pelo SINAN, com exceção da faixa etária e escolaridade que algumas categorias foram aglomeradas. Essa aglomeração foi baseada em inquéritos de saúde nacionais como a Pesquisa Nacional de Saúde (12) e Pesquisa Nacional de Saúde do Escolar (13), ambas realizadas pelo Instituto Brasileiro de Geografia e Estatística.

Todas as variáveis foram organizadas em um único banco de dados no Excel, onde as consistências internas das informações foram analisadas e, posteriormente, utilizadas para as análises estatísticas no software estatístico Stata (versão 14.2). 

### Métodos estatísticos

A tendência temporal das notificações de violência contra as mulheres foi calculada por meio da regressão linear de Prais-Winsten, que permitiu analisar as tendências de notificação como estáveis (p-valor>0,05), decrescentes (p-valor<0,05 e coeficiente de regressão negativo) ou crescentes (p-valor<0,05 e coeficiente de regressão positivo) (14). Esse coeficiente representa a variação anual, seja no período total de 2014 a 2023, ou no período mais recente de 2020 a 2023.

As variáveis dependentes foram compostas por dois indicadores: i) Taxa de incidência para cada tipo de violência (número absoluto das notificações de cada tipo de violência, dividido pelo total da população feminina residente, multiplicado por 100 mil), e ii) Proporção de cada tipo de violência (número absoluto das notificações de cada tipo de violência, dividido pela somatória das notificações por violência entre as mulheres, multiplicado por 100). Esses indicadores foram calculados para cada ano de interesse, e o ano foi a variável independente de ambos os modelos. 

As características sociodemográficas (faixa etária, escolaridade, raça/cor da pele, região geográfica) e as características da ocorrência (meios de agressão, relação de parentesco entre autor e vítima, local de ocorrência, encaminhamento no setor saúde e evolução do caso) foram analisadas por meio das frequências absolutas e relativas. 

## Resultados

As notificações por violência contra as mulheres apresentaram uma tendência crescente nos últimos 10 anos, com variação anual de 9,8 casos por 100 mil mulheres (p-valor<0,05). Essa tendência foi observada para todos os tipos de violência, com destaque para lesão autoprovocada (16,4 casos por 100 mil mulheres ao ano), violência financeira/econômica (11,9 casos por 100 mil mulheres ao ano) e violência sexual (10,8 casos por 100 mil mulheres ao ano). No período recente, a magnitude de aumento da violência contra as mulheres quase duplicou para 19,2 casos por 100 mil mulheres ao ano (p-valor<0,05). Esse cenário foi observado para quase todos os tipos de violência (Tabela 1). 

**Tabela 1:** Variação anual da taxa de incidência (por 100 mil mulheres) referente às notificações de violência contra as mulheres. Brasil, 2014–2023 (n=3.861.994)

Tipos de violência	Ano										Variação anual (Taxa/ano) (2014/2023)	Variação anual (Taxa/ano) (2020/2023)
	2014	2015	2016	2017	2018	2019	2020	2021	2022	2023		
Lesão autoprovocada	19,3	25,1	28,8	44,0	57,8	84,1	62,0	74,7	89,6	117,3	16,4ª	20,6ª
Violência física	94,9	101,5	110,0	133,6	140,9	152,2	120,0	123,3	142,3	192,2	6,2ª	16,0
Violência psicológica/moral	45,0	50,3	52,2	59,9	59,0	64,4	53,9	57,5	67,5	91,4	6,5ª	17,8ª
Violência tortura	4,1	4,7	5,0	6,4	5,5	5,9	5,0	5,1	5,9	7,5	4,5ª	14,0^a^
Violência sexual	23,6	23,5	26,1	31,3	34,6	37,7	32,5	38,6	46,4	63,3	10,8ª	21,6ª
Violência financeira/econômica	2,4	2,6	3,0	3,2	3,7	4,5	3,7	4,4	5,3	7,6	11,9ª	23,5ª
Violência negligência/abandono	12,9	14,1	15,7	19,7	20,6	21,2	16,7	20,3	23,1	32,3	8,6ª	15,2
Outras violências	11,7	15,2	17,6	28,0	42,7	58,0	45,7	51,0	65,1	91,1	18,6ª	23,7ª
Total	214,0	237,0	258,4	326,0	364,7	427,9	339,6	374,9	445,3	602,9	9,8ª	19,2ª

^a^Variação anual estatisticamente significativa (p-valor<0,05).

Mais de 220 mil notificações por violência contra as mulheres foram realizadas no Sistema de Informação de Agravos de Notificação de Violência Interpessoal e Autoprovocada em 2014, sendo que esse número aumentou cerca de 3 vezes em 10 anos, com quase 670 mil notificações realizadas em 2023 (Tabela 2). Do total de notificações, destacaram-se a violência física e a psicológica/moral ao longo do período analisado, com proporção média de 37,7% e 17,4%, respectivamente. Observou-se um aumento de 8,1% ao ano (p-valor<0,05) na proporção de notificação por lesão autoprovocada, enquanto a maioria dos demais tipos de violência apresentou redução na sua proporção, com destaque para tortura, violência sexual, e violência psicológica/moral (-5,6%; -4,1% e -4,0%/ano, respectivamente). No período mais recente (2020-2023), apenas os casos de tortura continuaram reduzindo (-4,9%/ano) (Tabela 2).

**Tabela 2:** Variação anual da proporção (%) das notificações de violência contra as mulheres. Brasil, 2014–2023 (n=3.861.994)

Tipos de violência	Ano										Variação anual (%) (2014/2023)	Variação anual (%) (2020/2023)
	2014	2015	2016	2017	2018	2019	2020	2021	2022	2023		
Lesão autoprovocada	9,0	10,6	11,2	13,5	15,8	19,7	18,3	19,9	20,1	19,5	8,1ª	1,9
Violência física	44,3	42,8	42,6	41,0	38,6	35,6	35,3	32,9	32,0	31,9	-4,1ª	-3,4
Violência psicológica/moral	21,0	21,2	20,2	18,4	16,2	15,0	15,9	15,3	15,2	15,2	-4,0^a^	-1,5
Violência tortura	1,9	2,0	1,9	2,0	1,5	1,4	1,5	1,4	1,3	1,2	-5,6ª	-4,9ª
Violência sexual	11,0	9,9	10,1	9,6	9,5	8,8	9,6	10,3	10,4	10,5	-0,3	2,7
Violência financeira/econômica	1,1	1,1	1,2	1,0	1,0	1,1	1,1	1,2	1,2	1,3	1,3	4,5ª
Violência negligência/abandono	6,0	6,0	6,1	6,0	5,6	5,0	4,9	5,4	5,2	5,4	-1,8	1,3
Outras violências	5,5	6,4	6,8	8,6	11,7	13,5	13,5	13,6	14,6	15,1	10,3ª	4,6ª
Total	220.324	246.201	270.747	344.397	388.486	459.495	367.574	408.850	489.123	666.797	-	-

^a^Variação anual estatisticamente significativa (p-valor<0,05).

De modo geral, as principais características sociodemográficas referentes às notificações foram: faixa etária de 20-59 anos, escolaridade de 4ª-8ª série do Ensino Fundamental incompleto, raça/cor da pele parda e da região Sudeste. Destaca-se o perfil sociodemográfico diferenciado para alguns tipos de violência, como: lesão autoprovocada (mulheres de raça/cor da pele branca), violência sexual (mulheres de 10-19 anos) e violência por negligência/abandono (mulheres com 9 anos ou menos). Ainda que a escolaridade de 4ª-8ª série incompleta tenha prevalecido no total de notificações, destaca-se que a escolaridade do Ensino Médio completo até Ensino Superior incompleto destacou-se para a maior parte dos tipos de violência analisados, com exceção de violência sexual e negligência/abandono (Tabela 3). 

**Tabela 3:** Frequência absoluta e relativa das notificações de violência contra as mulheres segundo características sociodemográficas. Brasil, 2014–2023 (n=3.861.994)

Características sociodemográficas	Lesão autoprovocada	Violência física	Violência psicológica/moral	Violência tortura	Violência sexual	Violência financeira/econômica	Violência negligência/abandono
	n (%)	n (%)	n (%)	n (%)	n (%)	n (%)	n (%)
**Faixa etária** (anos)							
≤9	5.810 (0,9)	58.375 (4,2)	41.427 (6,4)	3.724 (6,3)	102.764 (26,7)	1.407 (3,2)	115.887 (54,8)
10-19	211.003 (32,4)	262.655 (18,7)	116.558 (18,1)	11.747 (19,9)	177.278 (46,1)	2.879 (6,6)	42.737 (20,2)
20-59	416.503 (63,9)	1.022.481 (72,7)	447.794 (69,5)	40.837 (69,1)	100.275 (26,1)	30.546 (70,4)	13.724 (6,5)
≥60	16.829 (2,6)	60.866 (4,3)	38.090 (5,9)	2.648 (4,5)	4.059 (1,1)	8.468 (19,5)	38.517 (18,2)
Ignorado/branco	1.161 (0,2)	1.880 (0,1)	870 (0,1)	100 (0,2)	396 (0,1)	62 (0,1)	526 (0,2)
Escolaridade							
Até 4ª série do Ensino Fundamental incompleto	21.439 (3,3)	84.594 (6,0)	49.722 (7,7)	5.307 (9,0)	34.879 (9,1)	4.936 (11,4)	20.922 (9,9)
4ª até 8ª série do Ensino Fundamental incompleto	98.953 (15,2)	222.085 (15,8)	117.181 (18,2)	11.387 (19,3)	102.389 (26,6)	6.904 (15,9)	23.483 (11,1)
Ensino Fundamental completo até Ensino Médio incompleto	126.255 (19,4)	239.207 (17,0)	111.367 (17,3)	10.564 (17,9)	51.467 (13,4)	6.201 (14,3)	10.858 (5,1)
Ensino Médio completo até Ensino Superior incompleto	139.759 (21,5)	280.779 (20,0)	144.638 (22,4)	11.553 (19,6)	43.217 (11,2)	10.606 (24,5)	4.244 (2,0)
Ensino Superior completo	20.397 (3,1)	41.297 (2,9)	30.240 (4,7)	1.987 (3,4)	8.704 (2,3)	2.932 (6,8)	722 (0,3)
Ignorado/branco	244.503 (37,5)	538.295 (38,3)	191.591 (29,7)	18.258 (30,9)	144.116 (37,5)	11.783 (27,2)	151.162 (71,5)
**Raça/cor da pele**							
Branca	301.742 (46,3)	533.052 (37,9)	252.644 (39,2)	20.966 (35,5)	135.028 (35,1)	16.143 (37,2)	82.174 (38,9)
Preta	41.170 (6,3)	129.790 (9,2)	66.547 (10,3)	6.202 (10,5)	36.047 (9,4)	5.707 (13,2)	13.608 (6,4)
Amarela	5.688 (0,9)	11.388 (0,8)	5.349 (0,8)	582 (1,0)	3.191 (0,8)	462 (1,1)	1.214 (0,6)
Parda	244.570 (37,6)	588.119 (41,8)	273.570 (42,4)	27.633 (46,8)	180.189 (46,8)	18.595 (42,9)	89.659 (42,4)
Indígena	3.175 (0,5)	13.120 (0,9)	5.431 (0,8)	633 (1,1)	4.788 (1,2)	348 (0,8)	1.673 (0,8)
Ignorado/branco	54.961 (8,4)	130.788 (9,3)	41.198 (6,4)	3.040 (5,1)	25.529 (6,6)	2.107 (4,9)	23.063 (10,9)
**Região geográfica**							
Norte	25.708 (3,9)	81.087 (5,8)	55.716 (8,6)	5.534 (9,4)	55.065 (14,3)	2.702 (6,2)	9.661 (4,6)
Nordeste	101.621 (15,6)	231.662 (16,5)	115.151 (17,9)	12.857 (21,8)	71.032 (18,5)	13.909 (32,1)	42.704 (20,2)
Sudeste	314.387 (48,3)	785.711 (55,9)	321.805 (49,9)	27.418 (46,4)	153.739 (40,0)	16.759 (38,6)	73.130 (34,6)
Sul	148.516 (22,8)	218.968 (15,6)	115.776 (18,0)	8.343 (14,1)	69.001 (17,9)	7.881 (18,2)	65.370 (30,9)
Centro-Oeste	61.026 (9,4)	88.625 (6,3)	36.223 (5,6)	4.889 (8,3)	35.849 (9,3)	2.105 (4,9)	20.492 (9,7)
Ignorado/branco	48 (0,0)	204 (0,0)	68 (0,0)	15 (0,0)	86 (0,0)	6 (0,0)	34 (0,0)
Total	651.306 (100,0)	1.406.257 (100,0)	644.739 (100,0)	59.056 (100,0)	384.772 (100,0)	43.362 (100,0)	211.391 (100,0)

As características da ocorrência foram apresentadas nas tabelas 4 e 5. Os meios de agressão mais utilizados nos casos de violência interpessoal foram a força corporal/espancamento e a ameaça, já para os casos de lesão autoprovocada, o envenenamento/intoxicação (61,4%). Quanto ao parentesco entre autor da violência e vítima, destaca-se o cônjuge na tortura (29,6%), violência financeira/econômica (29,2%), na violência psicológica/moral (27,6%) e na violência física (26,2%); a mãe na violência por negligência/abandono (45,9%); e amigo(a)/conhecido(a) na violência sexual (23,1%); e a própria pessoa na lesão autoprovocada (94,9%) (Tabela 4). Esta proporção abaixo de 100% nos alerta para a possibilidade de sobreposição de tipos de violência nas fichas de notificação, ainda que o instrutivo do Ministério da Saúde oriente assinalar apenas o principal tipo de violência para fins de registro no Sistema de Informação de Agravos de Notificação de Violência Interpessoal e Autoprovocada (6). 

**Tabela 4:** Frequência absoluta e relativa das notificações de violência contra as mulheres segundo características das ocorrências. Brasil, 2014–2023 (n=3.861.994)

Características da ocorrência	Lesão autoprovocada	Violência física	Violência psicológica/moral	Violência tortura	Violência sexual	Violência financeira/econômica	Violência negligência/abandono
	n (%)	n (%)	n (%)	n (%)	n (%)	n (%)	n (%)
**Meios de agressão**							
Força corporal/espancamento	22.001 (3,2)	1.006.287 (54,4)	384.839 (42,1)	47.182 (38,9)	146.357 (41,7)	23.889 (33,4)	14.053 (9,6)
Enforcamento	25.996 (3,8)	100.122 (5,4)	55.481 (6,1)	14.925 (12,3)	12.503 (3,6)	5.332 (7,5)	1.446 (1,0)
Objeto contundente	9.660 (1,4)	88.422 (4,8)	35.006 (3,8)	7.501 (6,2)	6.504 (1,9)	3.350 (4,7)	2.786 (1,9)
Objeto perfuro cortante	109.183 (15,8)	164.026 (8,9)	46.356 (5,1)	10.756 (8,9)	12.759 (3,6)	3.546 (5,0)	2.805 (1,9)
Substância/objeto quente	6.708 (1,0)	11.791 (0,6)	4.108 (0,4)	1.426 (1,2)	1.687 (0,5)	510 (0,7)	6.157 (4,2)
Envenenamento/intoxicação	423.355 (61,4)	114.987 (6,2)	19.027 (2,1)	1.428 (1,2)	5.257 (1,5)	523 (0,7)	12.355 (8,5)
Arma de fogo	1.671 (0,2)	31.544 (1,7)	12.987 (1,4)	3.232 (2,7)	10.543 (3,0)	1.302 (1,8)	626 (0,4)
Ameaça	5.732 (0,8)	252.342 (13,6)	295.708 (32,4)	31.030 (25,6)	98.028 (27,9)	27.174 (38,0)	10.738 (7,3)
Outros	84.733 (12,3)	79.803 (4,3)	59.957 (6,6)	3.831 (3,2)	57.156 (16,3)	5.804 (8,1)	95.131 (65,1)
**Relação de parentesco entre autor e vítima**							
Pai	2.587 (0,4)	42.637 (3,1)	33.170 (5,0)	2.583 (4,1)	33.912 (8,8)	1.608 (3,4)	68.667 (24,6)
Mãe	4.270 (0,7)	42.215 (3,1)	26.345 (3,9)	2.300 (3,7)	7.008 (1,8)	1.852 (3,9)	128.012 (45,9)
Padrasto	580 (0,1)	20.181 (1,5)	18.310 (2,7)	1.852 (3,0)	35.963 (9,4)	484 (1,0)	4.116 (1,5)
Madrasta	167 (0,0)	3.696 (0,3)	2.170 (0,3)	268 (0,4)	727 (0,2)	115 (0,2)	905 (0,3)
Cônjuge	6.877 (1,1)	355.236 (26,2)	184.245 (27,6)	18.549 (29,6)	20.297 (5,3)	13.976 (29,2)	5.390 (1,9)
Ex-cônjuge	1.979 (0,3)	131.883 (9,7)	97.182 (14,5)	7.160 (11,4)	10.029 (2,6)	8.206 (17,2)	1.302 (0,5)
Namorado(a)	1.690 (0,3)	62.135 (4,6)	27.175 (4,1)	3.125 (5,0)	27.472 (7,2)	1.467 (3,1)	2.081 (0,7)
Ex-namorado(a)	696 (0,1)	38.556 (2,8)	25.198 (3,8)	2.113 (3,4)	6.843 (1,8)	1.753 (3,7)	572 (0,2)
Filho(a)	1.383 (0,2)	41.978 (3,1)	28.496 (4,3)	1.847 (2,9)	875 (0,2)	6.366 (13,3)	25.380 (9,1)
Irmão(ã)	1.299 (0,2)	45.480 (3,4)	20.432 (3,1)	1.389 (2,2)	8.413 (2,2)	1.448 (3,0)	4.536 (1,6)
Amigo(a)/conhecido(a)	3.723 (0,6)	146.703 (10,8)	64.733 (9,7)	5.813 (9,3)	88.648 (23,1)	1.975 (4,1)	4.771 (1,7)
Desconhecido(a)	2.101 (0,3)	119.447 (8,8)	42.203 (6,3)	8.022 (12,8)	77.095 (20,1)	2.151 (4,5)	2.512 (0,9)
Cuidador(a)	196 (0,0)	2.932 (0,2)	2.162 (0,3)	300 (0,5)	2.588 (0,7)	540 (1,1)	3.995 (1,4)
Patrão/chefe	134 (0,0)	2.001 (0,1)	2.984 (0,4)	178 (0,3)	1.577 (0,4)	279 (0,6)	173 (0,1)
Pessoa com relação institucional	657 (0,1)	6.816 (0,5)	5.011 (0,7)	310 (0,5)	3.772 (1,0)	168 (0,4)	1.450 (0,5)
Policial/agente da lei	463 (0,1)	5.113 (0,4)	1.946 (0,3)	376 (0,6)	681 (0,2)	155 (0,3)	169 (0,1)
Própria pessoa	620.663 (94,9)	193.445 (14,3)	29.073 (4,3)	2.731 (4,4)	1.512 (0,4)	585 (1,2)	9.632 (3,5)
Outros	4.712 (0,7)	95.706 (7,1)	57.601 (8,6)	3.761 (6,0)	56.458 (14,7)	4.685 (9,8)	15.303 (5,5)

A residência foi o local de ocorrência mais notificado para todos os tipos de violência, variando de 63,3% na violência sexual até 85,7% na lesão autoprovocada. Em relação ao encaminhamento para o setor de saúde e à evolução do caso, mais de 90% das notificações não tiveram respostas válidas (dados ignorados, branco ou não se aplica). Dentre os dados válidos, destaca-se o setor ambulatorial e a alta (Tabela 5).

**Tabela 5:** Frequência absoluta e relativa das notificações de violência contra as mulheres segundo características das ocorrências. Brasil, 2014–2023 (n=3.861.994)

Características da ocorrência	Lesão autoprovocada	Violência física	Violência psicológica/moral	Violência tortura	Violência sexual	Violência financeira/econômica	Violência negligência/abandono
	n (%)	n (%)	n (%)	n (%)	n (%)	n (%)	n (%)
**Local de ocorrência**							
Residência	558.159 (85,7)	931.295 (66,2)	478.196 (74,2)	42.481 (71,9)	243.382 (63,3)	37.087 (85,5)	141.009 (66,7)
Habitação coletiva	3.685 (0,6)	8.588 (0,6)	5.138 (0,8)	506 (0,9)	3.087 (0,8)	266 (0,6)	1.303 (0,6)
Escola	7.041 (1,1)	19.201 (1,4)	8.726 (1,4)	530 (0,9)	7.870 (2,0)	106 (0,2)	2.140 (1,0)
Local de prática esportiva	429 (0,1)	3.067 (0,2)	1.373 (0,2)	153 (0,3)	1.234 (0,3)	28 (0,1)	312 (0,1)
Bar ou similar	1.688 (0,3)	31.865 (2,3)	9.228 (1,4)	979 (1,7)	4.918 (1,3)	305 (0,7)	691 (0,3)
Via pública	21.577 (3,3)	192.572 (13,7)	67.107 (10,4)	7.931 (13,4)	41.717 (10,8)	2.518 (5,8)	11.542 (5,5)
Comércio/serviços	3.147 (0,5)	17.319 (1,2)	10.770 (1,7)	556 (0,9)	4.873 (1,3)	632 (1,5)	6.843 (3,2)
Indústrias/construção	257 (0,0)	1.276 (0,1)	752 (0,1)	126 (0,2)	902 (0,2)	42 (0,1)	66 (0,0)
Outros	12.814 (2,0)	52.153 (3,7)	32.930 (5,1)	3.521 (6,0)	37.566 (9,8)	1.349 (3,1)	25.573 (12,1)
Ignorado/branco	42.509 (6,5)	148.921 (10,6)	30.519 (4,7)	2.273 (3,8)	39.223 (10,2)	1.029 (2,4)	21.912 (10,4)
**Encaminhamentos setor saúde**							
Ambulatorial	11.771 (1,8)	42.907 (3,1)	18.365 (2,8)	2.008 (3,4)	13.680 (3,6)	811 (1,9)	4.186 (2,0)
Internação hospitalar	4.246 (0,7)	10.079 (0,7)	2.874 (0,4)	451 (0,8)	2.064 (0,5)	194 (0,4)	2.372 (1,1)
Ignorado/branco/não se aplica	635.289 (97,5)	1.353.271 (96,2)	623.500 (96,7)	56.597 (95,8)	369.028 (95,9)	42.357 (97,7)	204.833 (96,9)
**Evolução do caso**							
Alta	13.395 (2,1)	64.688 (4,6)	28.191 (4,4)	2.628 (4,5)	13.920 (3,6)	1.397 (3,2)	6.098 (2,9)
Evasão/fuga	361 (0,1)	981 (0,1)	376 (0,1)	41 (0,1)	195 (0,1)	30 (0,1)	937 (0,4)
Óbito por violência	334 (0,1)	764 (0,1)	81 (0,0)	49 (0,1)	28 (0,0)	2 (0,0)	51 (0,0)
Óbito por outras causas	25 (0,0)	48 (0,0)	21 (0,0)	6 (0,0)	7 (0,0)	7 (0,0)	63 (0,0)
Ignorado/branco	637.191 (97,8)	1.339.776 (95,3)	616.070 (95,6)	56.332 (95,4)	370.622 (96,3)	41.926 (96,7)	204.242 (96,6)

## Discussão

Este estudo evidenciou que as notificações de violência contra as mulheres apresentaram tendência de crescimento na última década, principalmente após a pandemia da covid-19. Dentre os tipos de violência, destaca-se o aumento das notificações de lesões autoprovocadas, violência sexual e financeira/econômica. Entretanto, proporcionalmente, destaca-se: i) violência física e psicológica/moral como principais tipos de violência contra as mulheres no Brasil, e ii) aumento dos casos de autolesão em detrimento dos casos de agressões interpessoais. Prevaleceram as notificações de mulheres adultas (20-59 anos), com escolaridade da 4ª-8ª série do Ensino Fundamental incompleto, e de raça/cor da pele parda. Dentre os casos de violência interpessoal destacaram-se como meio de agressão a força corporal/espancamento e a ameaça, entre os casos de lesão autoprovocada, o envenenamento/intoxicação. O cônjuge foi o principal agressor, e o ambiente domiciliar o principal local de ocorrência. 

O aumento dessas notificações pode ser reflexo de dois cenários: i) Aumento efetivo da violência contra as mulheres diante de uma construção histórica, cultural e social de uma sociedade machista, que perpetua até os dias atuais como principal obstáculo ao empoderamento feminino e a efetiva igualdade entre os gêneros (15,16); e ii) Aumento do registro das notificações por violência nos Sistemas de Informação em Saúde, seja devido às melhorias técnicas e operacionais (contemplando treinamento da equipe e informatização das Unidades) ou avanços na rede de apoio às mulheres (rede social e familiar, organizações da sociedade civil, equipamentos públicos). A Política Nacional de Enfrentamento à Violência contra as Mulheres de 2011 impulsionou esses avanços ao instituir uma rede de assistência que oferta atendimentos relacionados à saúde (como os Centros de Referências da Mulher), à assistência social (como as Casas Abrigos), à justiça (como os Núcleos da Mulher nas Defensorias Públicas e Juizados de Violência Doméstica) e à segurança (como as Delegacias Especializadas) (3,4,17).

Esse aumento das notificações quase que duplicou no período recente de análise, compatível com estudos nacionais e internacionais que apontam a pandemia da covid-19 como possível justificativa (18-21). Por um lado, diante das restrições de convívio social, observou-se o aumento da convivência entre a vítima e o potencial agressor, intensificando o nível de estresse, o consumo de bebidas alcoólicas e outras drogas, incertezas sobre o futuro e a redução de renda familiar. Por outro lado, ainda que o aumento da violência tenha sido detectado nos resultados do presente trabalho, a subnotificação deve ter sido intensificada considerando a desarticulação da rede de apoio e de proteção a essas mulheres no período de pandemia, com limitações de acesso à rede de atenção à saúde, e consequentemente, desencorajando as mulheres na efetivação da denúncia da violência (22,23). 

Dentre os tipos de violência, observou-se um aumento proporcional das notificações por lesões autoprovocadas em detrimento dos casos de violência interpessoal entre 2014 e 2023. A violência costuma desencadear e intensificar o processo de adoecimento às vítimas, seja ele físico e/ou psicoemocional, agudo e/ou crônico, de curto e/ou longo prazo, submetidas a tratamentos medicamentosos prolongados e/ou terapias (24). Esse adoecimento, por vezes, intensifica o ciclo de violência, podendo aumentar as taxas de suicídio e de lesões autoprovocadas (25). No Brasil, as lesões autoprovocadas acometem principalmente as mulheres, sendo o suicídio mais prevalente entre aquelas mais jovens (15-19 anos) (26).

Adicionalmente, analisar o perfil sociodemográfico das vítimas não só possibilita a identificação dos grupos mais vulneráveis à violência, como também a contextualização do perfil das mulheres com maior acesso à rede de cuidado, e, portanto, as mais notificadas. As mulheres adultas (20-59 anos), da 4ª-8ª série do Ensino Fundamental incompleto, da raça/cor da pele parda e da região Sudeste foi o perfil predominante para a notificação de violência, entretanto, não se pode afirmar que esse é o perfil das principais vítimas no Brasil. O Norte e Nordeste, por exemplo, costumam ser as regiões geográficas com maior subnotificação desses casos (27). Além disso, sabe-se que o perfil sociodemográfico das vítimas varia a depender do tipo de violência, mas de modo geral, quanto maior a vulnerabilidade da mulher, maior a chance de sofrer violência, e muitas vezes, não efetivar a notificação, por desconhecimento ou por medo (22). No presente estudo, a maior vulnerabilidade foi observada entre as notificações de violência sexual e de negligência/abandono. 

Os meios de agressão mais utilizados foram a força corporal/espancamento e a agressão nos casos de violência interpessoal, e o envenenamento/intoxicação nos casos de lesão autoprovocada. Vale ressaltar que a força corporal/espancamento tem sido cada vez menos utilizada em alguns tipos de violência, como é o caso da violência sexual (8), enquanto a ameaça tem sido um meio de agressão cada vez mais utilizado, principalmente quando as vítimas são mais jovens (10-19 anos), como foi o caso da violência sexual no presente estudo. Além da violência sexual, uma das maiores proporções de ameaça foi identificada na violência financeira/econômica e na violência psicológica/moral, onde predominou o cônjuge como principal autor e a residência como principal local de ocorrência. A violência doméstica costuma seguir um ciclo bem delimitado coexistindo ameaças e agressões físicas. Inicialmente, acontecem desentendimentos e provocações, seguidas de estratégias de ameaças como a separação e o impedimento da participação na vida dos filhos. E, por fim, a agressão física (28).

As mulheres vítimas de violência precisam ser acompanhadas por uma equipe multiprofissional e cercar-se de uma rede de apoio efetiva, no intuito não só de reduzir a rescindida da violência interpessoal, mas também de melhorar a qualidade de vida dessas mulheres e diminuir a chance de lesões autoprovocadas. Entretanto, mais de 90% das notificações não tiveram informações válidas sobre o encaminhamento do caso no setor saúde e sua evolução clínica. Essa ausência ou falhas no preenchimento das fichas de notificação podem ser reflexo do fato de que profissionais de saúde ainda compreendem a notificação como denúncia, dificultando a análise epidemiológica do problema (29). As unidades de atendimento de saúde são obrigadas a encaminhar as pacientes apenas em situações especiais, como é o caso de vítimas crianças ou adolescentes (Conselho Tutelar ou Ministério Público) e idosas (Conselho Municipal do Idoso ou Ministério Público). De modo geral, as equipes de saúde são orientadas a informar sobre a existência de serviços da rede de proteção social e sobre a importância da denúncia, mas não devem encaminhar o caso sem autorização explícita (30).

Contextualizar as notificações permite analisar o perfil de violência em que as mulheres estão submetidas, mas também fazer uma análise crítica sobre o processo de notificação do país. Esses sistemas devem não só fornecer informações de qualidade, como também, subsidiar melhorias práticas à população (5). Nesse sentido, potenciais limitações devem ser observadas na apreciação dos resultados do presente trabalho: i) a subnotificação dos casos de violência no Brasil; ii) o preenchimento parcial das fichas de notificação; e iii) a limitação para identificar os casos com sobreposição de violência (envolvendo mais de um tipo). Essa sobreposição dos tipos de violência limitou a interpretação de alguns resultados do manuscrito, como meios de agressão e relação de parentesco entre autor e vítima. Contudo, acredita-se que essas limitações não superam as potencialidades do estudo. Os dados são provenientes de um Sistema de Informação em Saúde regulamentado e consolidado em todo território nacional (6,7), permitindo o embasamento de discussões para o aperfeiçoamento dos Sistemas de Informação e melhorias para o Sistema Único de Saúde. Com o intuito de reduzir a limitação de subnotificação no sistema, instituiu-se como público de estudo o somatório das ocorrências por tipo de violência. Esse número foi maior que o total de notificações efetivamente realizadas (3.861.994 versus 2.503.337). 

Por fim, conclui-se que todos os tipos de violência contra as mulheres (lesão autoprovocada, violência física, psicológica/moral, tortura, sexual, financeira/econômica, negligência/abandono, outras) apresentaram aumento da sua notificação no país entre 2014 e 2023, com intensificação dessa magnitude nos último quatro anos (pós-pandemia da covid-19). Isso demonstra que o Brasil está na contramão de atingir a meta de “eliminar todas as formas de violência contra todas as mulheres e meninas nas esferas públicas e privadas no mundo até 2030”, conforme pactuado com a Organização das Nações Unidas por meio do objetivo 5 (Igualdade de Gênero) do Desenvolvimento Sustentável (31). As mulheres com 20-59 anos, da 4ª-8ª série do Ensino Fundamental incompleto, raça/cor da pele parda e da região Sudeste prevaleceram entre as notificações de violência. Entretanto, esse perfil foi diferente a depender do tipo de violência, destacando-se maior vulnerabilidade entre aquelas que sofreram violência sexual e negligência/abandono. A força corporal/espancamento e ameaça foram destacadas como principais meios de agressão, cônjuge da vítima como principal autor da violência, e a residência como principal local de ocorrência. Mais de 90,0% das informações referentes ao encaminhamento do caso no setor saúde e a evolução do caso não foram válidas. 

## Data Availability

O banco de dados e os códigos de análise utilizados na pesquisa estão disponíveis em https://datasus.saude.gov.br/>.
